# Inositols affect the mating circadian rhythm of *Drosophila melanogaster*

**DOI:** 10.3389/fphar.2015.00111

**Published:** 2015-06-05

**Authors:** Kazuki Sakata, Haruhisa Kawasaki, Takahiro Suzuki, Kumpei Ito, Osamu Negishi, Takuo Tsuno, Hiromi Tsuno, Youta Yamazaki, Norio Ishida

**Affiliations:** ^1^Ishida Group of Clock Genes, Biomedical Research Institute, National Institute of Advanced Industrial Science and TechnologyTsukuba, Japan; ^2^Graduate School of Life and Environmental Sciences, University of TsukubaTsukuba, Japan; ^3^SHIGRAY Inc.Sumida, Japan; ^4^Faculty of Life and Environmental Sciences, University of TsukubaTsukuba, Japan; ^5^Tsuno Food Industrial Co. Ltd.Wakayama, Japan

**Keywords:** *Drosophila melanogaster*, circadian rhythm, ice plant, *myo*-inositol, mating succession

## Abstract

Accumulating evidence indicates that the molecular circadian clock underlies the mating behavior of *Drosophila melanogaster.* However, information about which food components affect circadian mating behavior is scant. The ice plant, *Mesembryanthemum crystallinum* has recently become a popular functional food. Here, we showed that the close-proximity (CP) rhythm of *D. melanogaster* courtship behavior was damped under low-nutrient conditions, but significantly enhanced by feeding the flies with powdered ice plant. Among various components of ice plants, we found that *myo*-inositol increased the amplitude and slightly shortened the period of the CP rhythm. Real-time reporter assays showed that *myo*-inositol and D-pinitol shortened the period of the circadian reporter gene *Per2-luc* in NIH 3T3 cells. These data suggest that the ice plant is a useful functional food and that the ability of inositols to shorten rhythms is a general phenomenon in insects as well as mammals.

## Introduction

The physiology and behavior of many organisms can adapt to daily and seasonal environmental changes via circadian clocks that comprise an endogenous self-sustained timekeeping system (Dunlap, 1999). Furthermore, the molecular mechanisms of circadian clock genes that consist of transcriptional–translational feedback loops are conserved from flies to humans (Kako and Ishida, 1998). A core oscillator mechanism of circadian rhythm and feedback loops involving several clock genes such as including *period* (*per*) control locomotor activity and eclosion of the fruit fly, *Drosophila melanogaster* (Dunlap, 1999). The relationships between behavioral rhythms and circadian clock genes have been studied in mutants of this fly with defective feedback loops.

Accumulating evidence indicates that the circadian clock underlies the reproductive behavior of *D. melanogaster* (Beaver and Giebultowicz, 2004; Kadener et al., 2006). The circadian rhythm of mating succession is controlled by the clock genes, *per* and *tim* in *Drosophila* (Sakai and Ishida, 2001). Heterosexual fly couples exhibit significantly different circadian activity from individual flies, having a brief rest phase around dusk followed by activity throughout the night and early morning (Fujii et al., 2007); this is referred to as the close-proximity (CP) rhythm. Analyses of CP rhythms have shown that circadian clocks regulate male courtship behavior in a circadian manner and that a core component of circadian clock, *per*, is regulated to generate CP rhythms. We previously identified the brain clock neurons that are responsible for the circadian rhythms of the CP behavior that reflects male courtship motivation under normal nutrient conditions (Hamasaka et al., 2010). However, how low-nutrient foods (LNFs) affect *Drosophila* circadian CP behavioral rhythms remains unknown.

A recent study found that inositol synthesis is involved in maintaining the period of circadian behavior in mice (Ohnishi et al., 2014), suggesting that dietary inositol affects the circadian rhythm of CP behavior. Furthermore, inositol is useful against depression (Mukai et al., 2014; Zhao et al., 2015). The African ice plant, *Mesembryanthemum crystallinum*, is abundant in inositols that are known to promote health (Lee et al., 2014). Here, we found that powdered ice plant gradually increased the CP behavior of *D. melanogaster* under low-nutrient conditions. Furthermore, adding inositol to the diet slightly shortened the period of the *Drosophila* CP rhythm. We also found that inositols concentration-dependently shortened the circadian rhythms of clock gene expression in mammalian NIH3T3 cells. These findings when taken together indicate that the ability of inositols to shorten these rhythms is a general phenomenon in animals regardless of species.

## Materials and Methods

### Food Composition

Boiled standard medium consisting of 8% corn meal, 5% glucose, 5% dry yeast extract, 0.64% agar was supplemented with 0.5% propionic acid and 0.5% butyl p-hydroxybenzoate (standard food, SF). Designated LNF comprising 5% glucose, 1.5% agar, 0.5% butyl *p*-hydroxybenzoate was supplemented without (LNF) or with (LNFI) 0.5% ice plant powder (Nihon Advanced Agri Corporation, Nagahama, Shiga, Japan).

### Separation of Inositols in Ice Plant

*Myo*-inositol and pinitol that have similar structures were separated from ice plant powder by high-performance anion exchange chromatography (HPAE-PAD) using a column containing Dinox CarboPac MA1 (Negishi et al., 2015).

### Fly Strains

The wild-type strain, Oregon-R and the clock mutant *per*^0^ were raised under a 12-h light/12-h dark cycle at 25°C on SF.

### Close-Proximity Assays

About 40 male and female flies were maintained in vials with SF for 3 days starting from the third day after eclosion. One male and one female from the same genotype were lightly anesthetized with CO_2_ and rapidly placed in 35-mm-diameter dishes containing SF or LNF. The dishes were then mounted under a CCD camera, (Watec Co. Ltd., Yamagata, Japan) which is sensitive to light at the near infra-red range and a recording system was established as described (Fujii et al., 2007; Hamasaka et al., 2010). A fluorescent lamp provided illumination at 100 lux and a red LED provided constant dim light <1 lux. Time-lapse images (one frame per 10 s) were sent to a personal computer. The locations of the flies on the *X* and *Y*-axes of the images were determined using ImageJ Plugin (http://rsb.info.nih.gov/ij/). The CP index of each pair was calculated from the *X–Y* value with a threshold (<5 mm) between them. Male flies moving to within 5 mm of a female and those remaining >5 mm from a female were scored as 1 or 0, respectively, in the algorithm of the CP index program. All CP assays proceeded with flies of the same genotypes and the data were averaged for each genotype. The circadian rhythmicity of CP was determined using autocorrelation (CORREL function) analysis (Levine et al., 2002). The free-running period and the power of rhythmicity in each genotype were calculated as the average of the free-running period and the maximum correlation between each pair evaluated by autocorrelation as being rhythmic (CORREL function; Hamasaka et al., 2010).

### Statistical Analysis

All data are expressed as means ± SEM and were statistically evaluated using Student’s *t*-test for single comparisons and one-way ANOVA. *P* < 0.05 was considered to indicate a statistically significant difference.

### Cell Culture

NIH3T3 cells were incubated in Dulbecco’s modified Eagle’s medium (D-MEM) supplemented with 10% fetal bovine serum and a mixture of penicillin and streptomycin at 37°C under a humidified 5% CO_2_ atmosphere.

### Real-Time Luciferase Assays

The *Per2* promoter regions were cloned into pGL3-dLuc (Ohno et al., 2007), and then reporter plasmids (2 μg) were transfected into NIH3T3 cells (35-mm collagen type I-coated dishes) using HilyMax (Dojindo Laboratories, Kumamoto, Japan). The cells were stimulated with 100 nM dexamethasone (Sigma–Aldrich) for 2 h in serum-free Dulbecco’s MEM and then the medium was replaced with fresh Dulbecco’s MEM containing 100 μM luciferin (Wako Pure Chemical Industries), 25 mM HEPES (pH 7.2), and 10% fetal bovine serum. Bioluminescence was measured and integrated for 1 min at intervals of 10 min using an LM-2400 photon detection unit (Hamamatsu Photonics, Hamamatsu, Japan). The cells were cultured in a luminometer for 3 days to evaluate bioluminescence. Reporter gene expression was detrended by subtracting an average of 12 h from the raw data. Peaks and troughs were measured on detrended charts using a scale to calculate the phase of reporter-gene expression. The average period (hours) between peaks was calculated from detrended data accumulated for >5 days.

## Results

### Feeding with Ice Plant Powder Enhanced CP Rhythm of *Drosophila* Courtship Behavior

**FIGURE 1:**
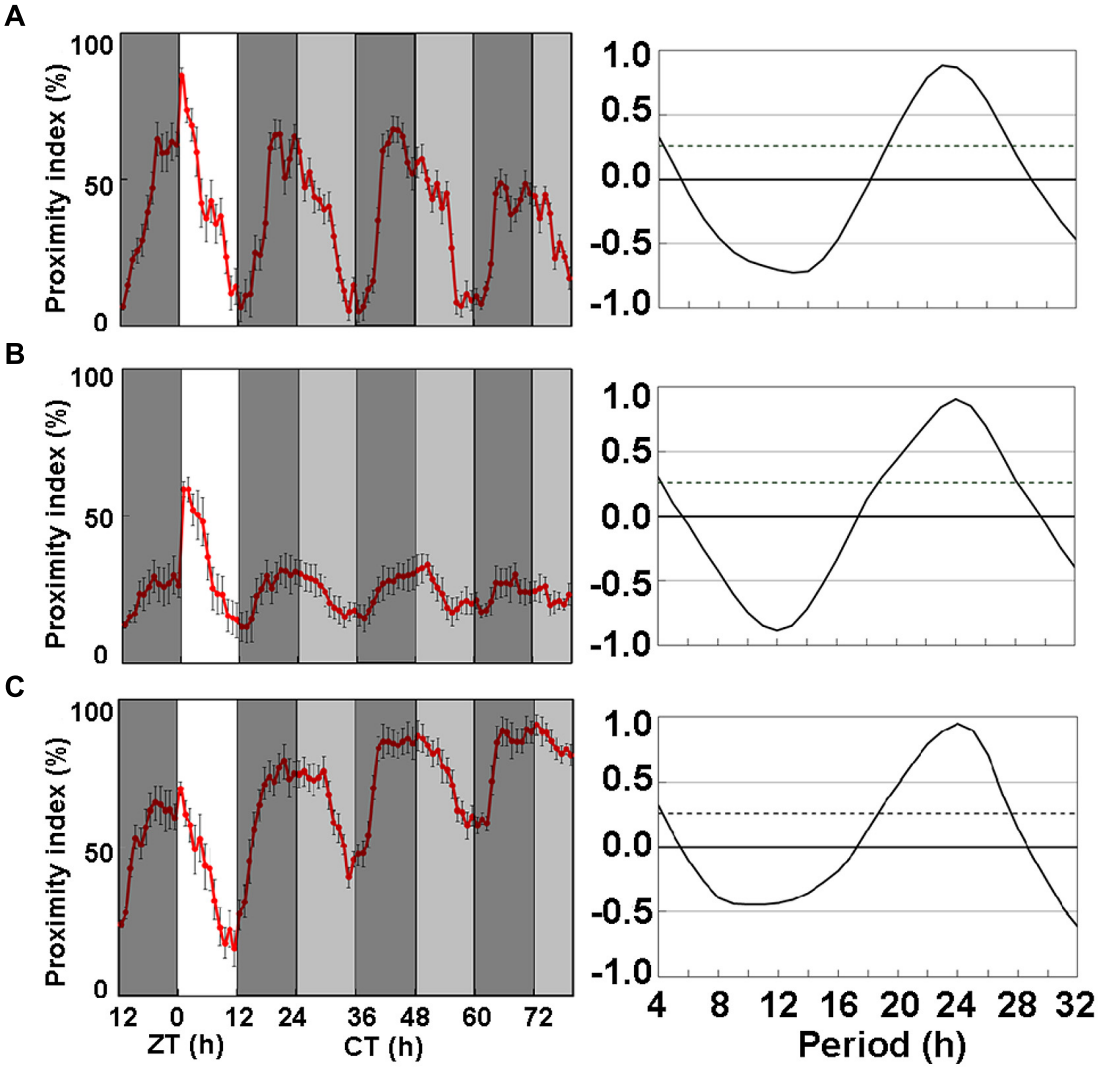
**Close-proximity (CP) rhythms of wild-type *Drosophila melanogaster* strain, Oregon-R on three types of medium. (A)** Proximity index shows obvious circadian rhythms in Oregon-R. Flies were paired at dusk during LD 12:12 cycle. Data were obtained under constant darkness (DD) after 24 h under LD 12:12. Pairs of Oregon-R flies exhibited daily CP behavior under LD 12:12. Rhythmic CP behavior persisted under DD on **(A)** Standard food (SF; *n* = 7), **(B)** LNF (*n* = 27), and **(C)** LNF containing 0.5% ice plant powder (LNFI; *n* = 21). All CP rhythms were statistically tested by autocorrelation (CORREL function) analysis (right panels), resulting in significant circadian rhythmicity (95% significance indicated by dotted line). The amplitude of CP rhythm decreased on flies fed with low-nutrient food (LNF). White area on graph indicates day; black and gray bars indicate subjective night and subjective day, respectively. Data from 7 to 27 pairs were averaged for each panel. Black error bars indicate SEM.

The CP rhythms of heterosexual pairs of Oregon-R flies dipped at dusk under LD12:12 as described (Fujii et al., 2007; Hamasaka et al., 2010) and persisted under DD with a dip at subjective lights-off (CT12; Hamasaka et al., 2010). The circadian CP rhythm of Oregon-R fed with SF was obvious (**Figure 1A**) and the amplitude and period were very similar to those previously reported (Hamasaka et al., 2010). To understand the effect of LNF on CP rhythms, we examined the CP rhythms of Oregon-R flies on LNF without cornmeal and yeast extract (**Figure 1B**). The amplitude of the CP rhythm declined under DD after a light and dark (LD12:12) cycle. Among several compounds that we screened for the ability to recover the amplitude of the CP rhythm under LNF, that LNFI sequentially promoted the activity of the rhythm (**Figure 1C**). Thus, ice plant powder contains candidate substances that might recover the amplitude of the CP rhythm under LNF.

### Rhythmicity of *Drosophila* Courtship Behavior Requires the Clock Gene *Period*

**FIGURE 2:**
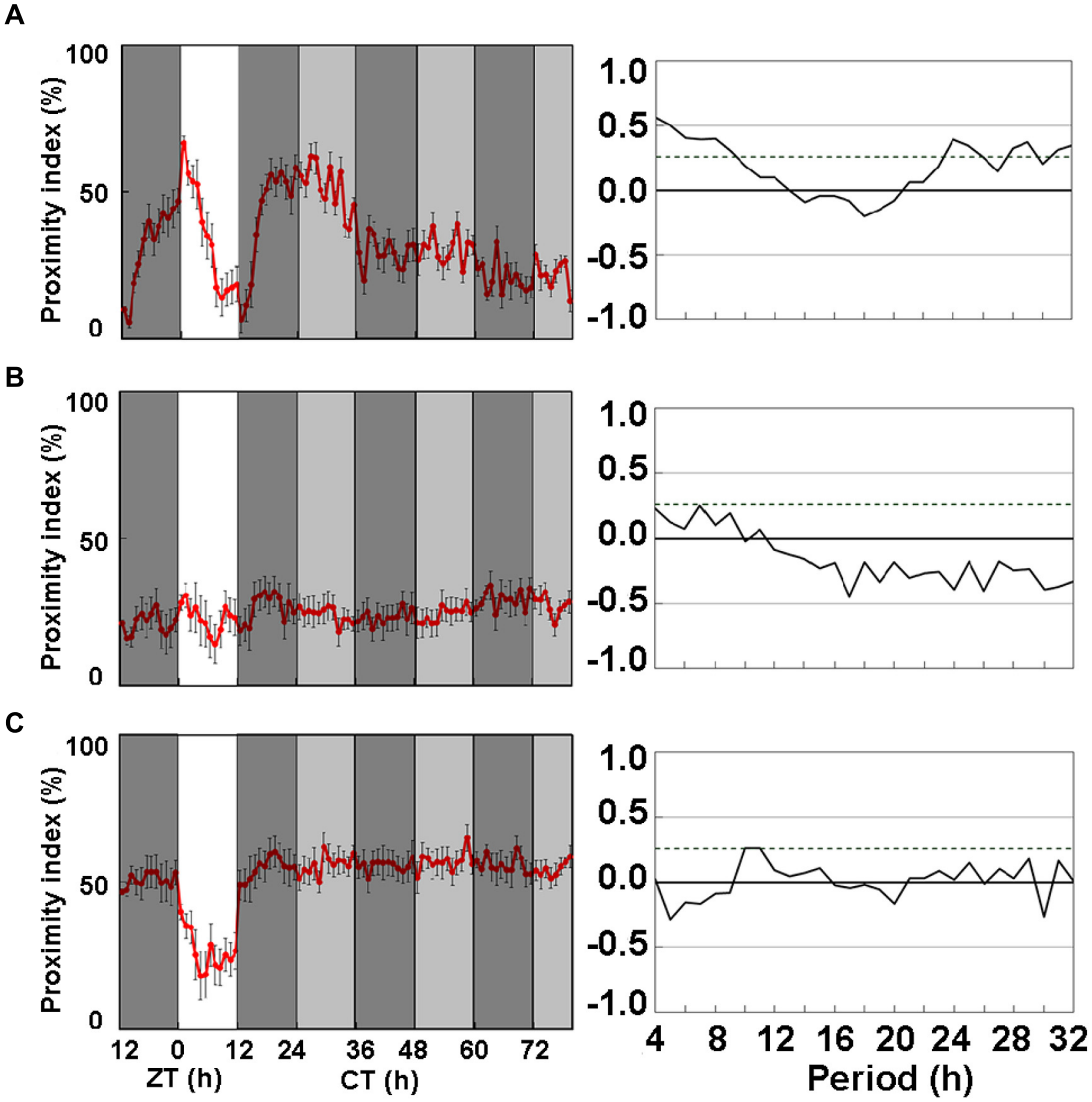
**Close-proximity rhythms of *D. melanogaster* mutant, *per*^0^, on three types of medium.** Proximity index shows arrhythmia in mutant strain *per*^0^ under constant darkness (DD). Data were obtained under DD after 24 h under LD 12:12. Pairs of *per*^0^ flies exhibited daily CP behavior under LD 12:12. Arrhythmic CP behavior persisted under DD on **(A)** SF (*n* = 8), **(B)** low nutrient food (LNF; *n* = 10), and **(C)** LNF containing 0.5% ice plant powder (LNFI; *n* = 10). All CP findings were statistically tested by autocorrelation (CORREL function) analysis (right panels), resulting in non-circadian rhythmicity (95% significance indicated by dotted line). White area on graph indicates day; black and gray bars indicate subjective night and subjective day, respectively. Data from 8 to 10 pairs are averaged for each panel. Black error bars indicate SEM.

Since ice plant powder promoted the activity and amplitude of CP rhythms in wild-type flies, we investigated the effects of LNFI on *period* mutant, *per*^0^ flies. **Figures 2A–C** shows the CP rhythms of *per*^0^ mutants fed with SF, LNF, and LNFI, respectively. The CP rhythms of *per*^0^ heterosexual pairs of flies dipped at dusk under LD, but became arrhythmic under DD for 2 days (**Figure 2A**) and in flies fed with LNF (**Figure 2B**). However, LNFI significantly enhanced the activity of the CP rhythm (**Figure 2C**). These data indicate that rhythmicity of CP behavior requires the *period* gene and that ice plant powder includes a promoter of CP rhythmic activity.

### *Myo*-Inositol Shortens Circadian Period of CP Rhythms and Activates the Amplitude of CP Behavior

**FIGURE 3:**
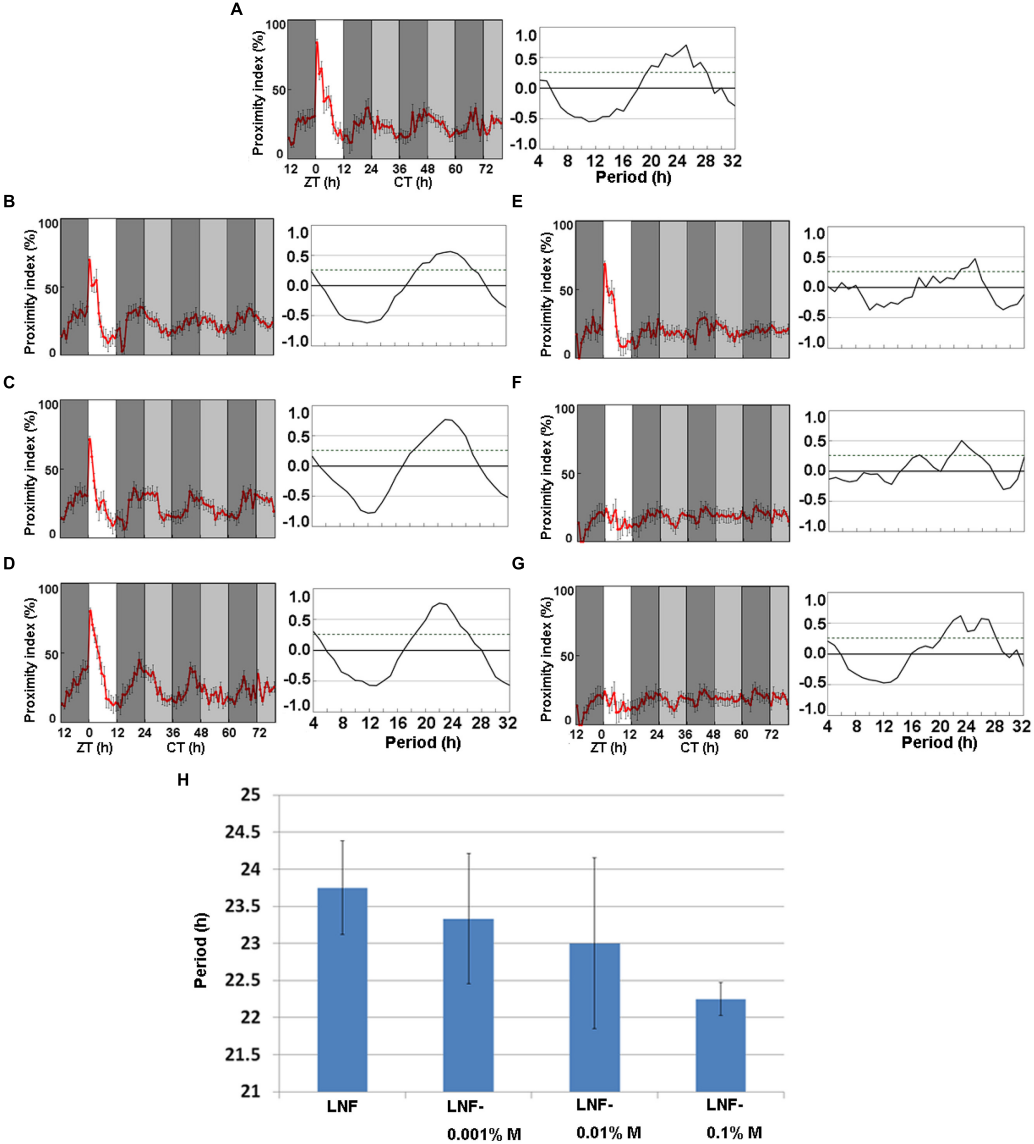
**Close-proximity rhythms of wild-type *D. melanogaster* strain, Oregon-R on **(A)** LNF and LNF including **(B)** 0.001%, **(C)** 0.01%, and **(D)** 0.1% *myo*-inositol and **(E)** 0.001%, **(F)** 0.01 **(G)** and 0.1% D-pinitol. (H)** Period of CP rhythms at different *myo*-inositol concentrations in LNF. Proximity index shows obvious circadian rhythms on *myo*-inositol mixed low nutrient food. Flies were paired at dusk during LD 12:12 cycle. Data were obtained under constant darkness (DD) after 24 h under LD 12:12. Pairs of Oregon-R flies exhibited daily CP behavior under LD 12:12. Rhythmic CP behavior persisted under DD on **(A)** LNF, **(B)** LNF-0.001% M, **(C)** LNF-0.01% M, **(D)** LNF-0.1% M (all *n* = 5). Arrhythmic CP behavior persisted under DD on **(E)** LNF including D-pinitol 0.001% (*n* = 4), **(F)** 0.01% (*n* = 5), and **(G)** 0.1% (*n* = 5). All CP data were statistically tested using autocorrelation (CORREL function) analysis (right panels), resulting in significant circadian rhythmicity (95% significance indicated by dotted line). White area on graphs indicates day; black and gray bars indicate subjective night and subjective day, respectively. Data from five pairs were averaged for each panel. Black error bars indicate SEM. **(H)** Period length of CP rhythms of wild-type *D. melanogaster* strain, Oregon-R on **(A)** LNF (23.75 h), LNF containing **(B)** 0.001% (23.33 h), **(C)** 0.01% (23 h), and **(D)** 0.1% (22.25 h) *myo*-inositol. Differences among these period lengths were not significant (Student’s *t*-test). LNF, low-nutrient food; M, *myo*-inositol.

**Figure 1** indicates that ice plant powder contains substances that promote the activity of CP rhythm. We therefore separated low-molecular weight substances in ice plant powder using HPLC and found 4.5 and 51.4 mg of *myo*-inositol and D-pinitol/g of fresh weight, respectively. We examined the effects of LNF containing either *myo*-inositol (**Figures 3B–D**) or D-pinitol (**Figures 3E–G**) at concentrations of 0.001, 0.01, and 0.1% each on the wild-type strain, Oregon-R to determine whether they are involved in promoting the activity of CP rhythms. **Figure 3A** shows the CP rhythms of heterosexual pairs of flies fed with LNF and **Figures 3B–D** shows that 0.001, 0.01, and 0.1% *myo*-inositol slightly promoted the amplitude of the CP rhythm, whereas the effects of the respective tested concentrations of D-pinitol did not significantly differ (**Figures 3E–G**). *Myo*-inositol seemed to dose-dependently shorten the period of CP rhythms (**Figure 3H**). These data suggest that *myo*-inositol not only increases the amplitude, but also shortens the phase of CP rhythms.

### *Myo*-Inositol Shortened Per2-luc Oscillation Period in Mammalian Cultured Cells

**FIGURE 4:**
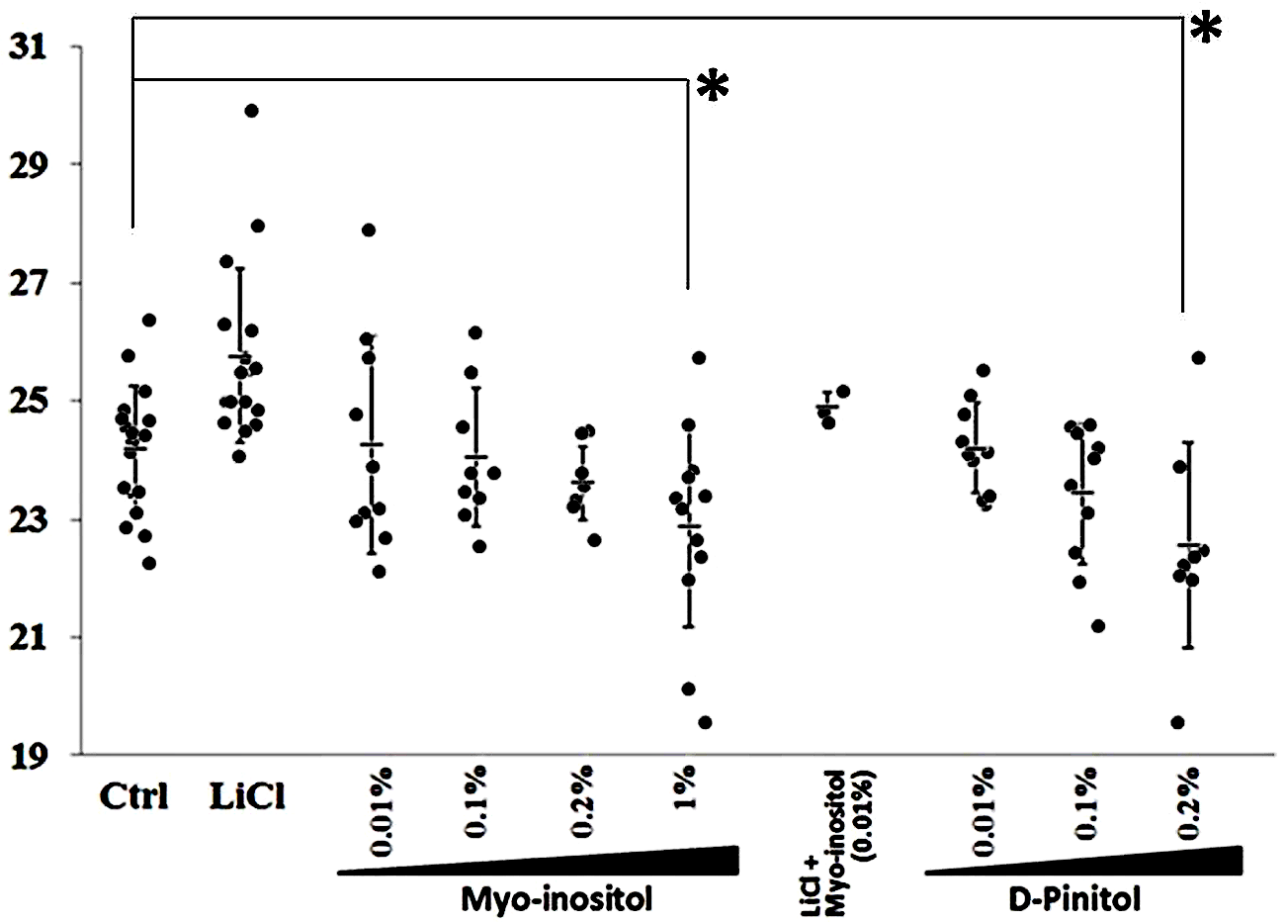
**Inositols at various concentrations shorten period of Per2-luc reporter gene oscillation in mammalian cultured cells.** Expression of Per2-luc reporter gene introduced into NIH 3T3 cells determined using real-time reporter assays. Mean estimated expression period was 24.2 h (Control). Adding lithium chloride (LiCl; positive control) lengthened period of Per2-luc expression. Data are shown as mean ± SEM. Significant difference between control and treated cells (^∗^*p* < 0.05, *t*-test).

We investigated the effects of *myo*-inositol on the period of reporter gene expression driven by Per2 in NIH 3T3 cells to determine whether it affects the phase of CP rhythms in mammals (**Figure 4**). Increasing *myo*-inositol concentrations tended to shorten the period of the CP rhythm, and 1% *myo*-inositol significantly shortened the period. D-pinitol (0.2%) also shortened the period of Per2-luc oscillation in cultured NIH 3T3 cells. Thus, inositols not only shortened the period of *Drosophila* behavior but also the period of mammalian cells.

## Discussion

We showed that the amplitude of CP rhythms was significantly reduced in wild-type flies fed with LNF. In contrast, LNF containing 0.5% ice plant powder (LNFI) recovered the amplitude of CP rhythm in these flies and the rhythm gradually became robust and high at tough. These findings suggested that ice plant powder contains substances that promote CP activity.

We analyzed inositol contents in ice plants using HPLC. The ice plants (100 g) grown in plant factory contained 51.4 mg of D-pinitol and 4.5 mg of *myo*-inositol and we analyzed the effects of these inositols upon the amplitude of CP rhythm. *Myo*-inositol increased the amplitude of CP rhythm in *Drosophila*, whereas D-pinitol did not. Therefore, we postulated that nutrients missing from LNF such as yeast extract and corn meal might contain *myo*-inositol. In fact, 5% dry yeast extract in the SF contained 1.07-mM *myo*-inositol and 758-nM D-pinitol, and 0.5% ice plant powder contained 991-μM *myo*-inositol and 14.2-μM D-pinitol. Thus, yeast extract in SF contains a sufficient amount of *myo*-inositol to promote the CP rhythm.

*Myo*-inositol slightly reduced the period of the CP rhythm, but it did not affect either the amplitude or the period of the locomotor rhythm (Supplementary Figure S1). This suggests that the CP rhythm might be one output for the circadian clock or that *myo*-inositol is involved in mating biochemistry (Papaleo et al., 2009; Colone et al., 2010).

The CP behavior under DD in *per*^0^ circadian clock mutant was arrhythmic, indicating that this rhythm required the molecular circadian clock. However, ice plant powder constantly enhanced the CP rhythm in this mutant, suggesting that it contains unknown factors that promote the CP behavior without affecting circadian rhythms in both *per*^0^ mutant and wild-type Oregon-R flies.

We studied the CP rhythms of Oregon-R flies in LNF containing 0.001, 0.01, and 0.1% *myo*-inositol (55.5 μM, 555 μM, and 5.55 mM, respectively) or D-pinitol (51.5 μM, 515 μM, and 5.15 mM, respectively). *Myo*-inositol at 0.01 and 0.1% promoted the amplitude, and dose-dependently shortened the period of the CP rhythm. Considering the recent suggestion that *myo*-inositol is required to maintain the period of circadian behavior in mice (Ohnishi et al., 2014), it appears to be common circadian regulator among various species. Otherwise, promoting the amplitude and the period of CP rhythms did not significantly differ among flies fed with LNF containing different ratios of D-pinitol. Although *myo*-inositol increased the amplitude of the CP rhythm, D-pinitol had no effect despite having a similar chemical structure to that of *myo*-inositol. However, both *myo*-inositol and D-pinitol shortened the period of mammalian cells, indicating that D-pinitol exerts different effects upon *Drosophila* and mammals. *Myo*-inositol shortened the male CP rhythm and increased the amplitude of the rhythm. Considering with that inositols are used to treat depression(Mukai et al., 2014; Zhao et al., 2015), the CP rhythm assay of *Drosophila* might be useful for screening drugs to treat depressive disorders in future.

## Conclusion

The CP behavior under DD in the *per*^0^ mutant was arrhythmic, indicating a requirement for the molecular circadian clock gene *period*. Ice plant powder enhanced the CP activity of the *per*^0^ mutant without recovering rhythmicity. These data suggest that ice plant powder has unknown factors that promote the activity of CP behavior without affecting the circadian rhythm in *Drosophila*. The ice plant component *myo*-inositol increased the proximity index but also slightly shortened the period of CP rhythm in *Drosophila*. Exogenous inositols concentration-dependently shortened the period of the circadian oscillation rhythm of the *mPer2-luc* reporter in cultured mammalian NIH3T3 cells. The ability of inositols to shorten rhythms might be a general feature of insects as well as mammals.

## Conflict of Interest Statement

The authors declare that the research was conducted in the absence of any commercial or financial relationships that could be construed as a potential conflict of interest.

## Supplementary Material

**Figure S1 | Myo-inositol did not affect the locomotor activity rhythm of Drosophila fed with low-nutrient food. Locomotor activity was analyzed as described (Inoue et al., 2002). Data are shown as means ± SEM. No significant difference among these period lengths (Student’s *t*-test)**.
