# Supplementary figures and images for: Inositols affect the mating circadian rhythm of Drosophila melanogaster
Source: Front Pharmacol. 2015 Jun 5;6:111. doi: 10.3389/fphar.2015.00111 (PMC4456571; doi:10.3389/fphar.2015.00111)

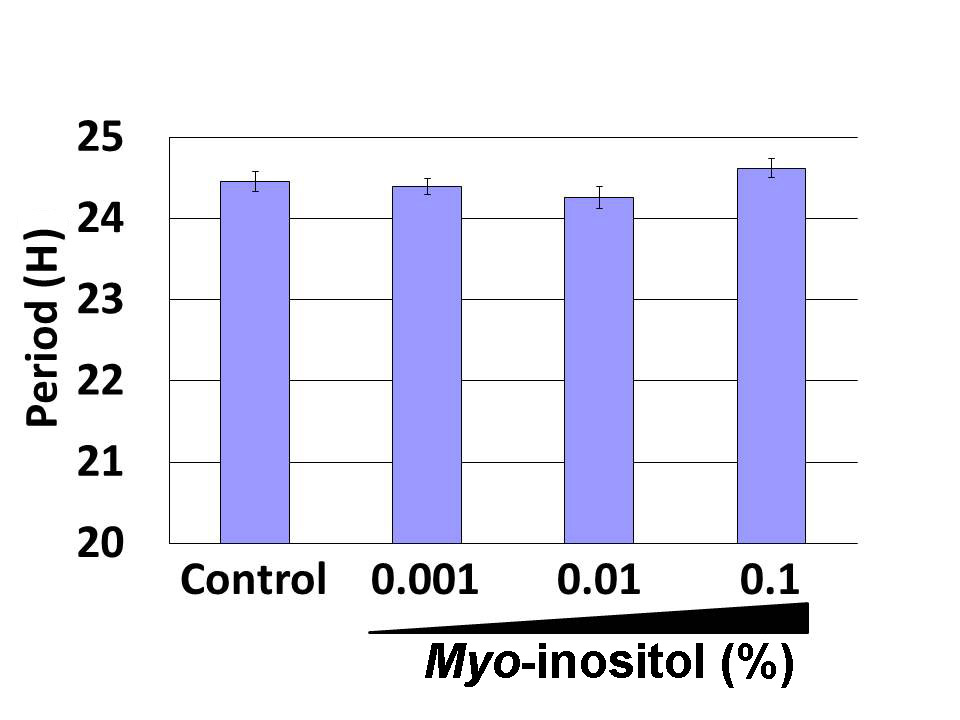

Supplement: Supplementary file 1 [file Image_1.JPEG]
